# Positive Guidance Effect of Ideological and Political Education Integrated With Mental Health Education on the Negative Emotions of College Students

**DOI:** 10.3389/fpsyg.2021.742129

**Published:** 2022-01-25

**Authors:** Xueting Li, Yanhong Gao, Yanfeng Jia

**Affiliations:** ^1^The School of Humanities and Social Sciences, Guangzhou Civil Aviation College, Guangzhou, China; ^2^School of Psychology, Fujian Normal University, Fuzhou, China; ^3^Counseling and Psychological Services, South China University of Technology, Guangzhou, China; ^4^Mental Health Education and Counseling Center, Guangzhou College of South China University of Technology, Guangzhou, China

**Keywords:** ideological and political, college students’ negative emotions, mental health, intervention experiment, positive guidance, entrepreneurial behavior

## Abstract

This exploration aims to solve the problems of imperfect psychological health education system and poor educational effects on college students. Here, ideological and political education is integrated with mental health education to investigate the role of collaborative intervention in guiding college students to resist negative emotions. First, an overview is offered of research on ideological and political education, mental health education, and negative emotions by the literature survey method. Moreover, a comprehensive investigation is also conducted on research objects, through the questionnaire, to understand the current situation of negative emotions of college students. And finally, an intervention experiment is taken on the negative emotions of college students by combining ideological and political education with mental health education. The results show that after 10 weeks of intervention experiment by combining ideological and political education with mental health education, there are significant differences in depression, negative emotions, somatization symptoms, and interpersonal problems between the treatment group and the control group (*P* < 0.01). Besides, there are significant differences in depression, negative emotions, somatization symptoms, and interpersonal problems between sophomores in the treatment group and the control group (*P* < 0.01). Moreover, there are significant differences in depression, negative emotions, somatization symptoms, and interpersonal problems between male participants in the treatment group and the control group (*P* < 0.01). In summary, ideological and political education integrated with mental health education has a positive guidance effect on the negative emotions of college students, greatly improving the negative emotions of the students, helping the students to regulate their emotions, and benefiting their study and life a lot. The purpose of integrating ideological and political education with mental health education is to provide reference for refining the mental health education system of college students and strengthening the positive guidance of negative emotions of college students.

## Introduction

Last year, the outbreak of corona virus disease 2019 (COVID-19) across China caused tens of thousands of people infected and huge property losses, besides anxiety and panic among the public inducing many psychological problems (Li et al., 2020). Uncertain factors such as ignorance, difficulties in control, and unpredictability of the epidemic may be the main psychological reasons. More and more studies have proven that intolerance of uncertainty is an important factor in the generation and maintenance of emotional disorders (Hebert and Dugas, 2019; Hwang et al., 2020). For college students with highly intolerable uncertainty, the uncertain situations or events encountered can cause them to produce more negative emotions, affecting their physical and mental health. Meanwhile, the social anxiety and negative problems are serious among college students (Tao et al., 2017; Huang et al., 2020; Tindall and Curtis, 2020).

The common methods to adjust negative emotions of college students mainly include drug treatment, cognitive intervention, and exercise (Kiosses et al., 2017; Szanto, 2017). Goranitis et al. (2017) conducted a genome-based trial of anti-negative drug treatment using nortriptyline and escitalopram, combined with cognitive behavioral therapy (CBT) for patients with moderate negative disorders to effectively improve their anxiety and negative emotions (Goranitis et al., 2017). Vîslă et al. (2018) confirmed the effectiveness of CBT in groups in improving anxiety through collective CBT for people with social anxiety disorder (Vîslă et al., 2018). Glowacki et al. (2017) arranged for patients with negative symptoms to take physical exercises for 8 weeks to activate their emotional state and ultimately alleviating their negative emotions (Glowacki et al., 2017). Cai et al. (2017) implemented cognitive emotion regulation on the mentality and behavior of undergraduates with Hepatitis B virus generally under psychological stress. They found that cognitive emotion regulation can bring a positive mental state, reduce stress, and correct problem behavior (Cai et al., 2017). To sum up, certain intervention measures for college students with negative emotions can help them free themselves from the existing negative state and restore health and vitality as soon as possible. In addition, mental health education is particularly critical, which is the elementary and pivotal link in the prevention and treatment of all psychological diseases. However, mental health education was started late in China, and it has not formed a comprehensive system yet. Nevertheless, due to its special position in education, it soon stimulated the enthusiasm of the majority of scholars and initially formed the fundamental theoretical results.

Ideological and political education has accumulated rich results after years of theoretical research and specific practice, and there is a relatively perfect education system at present. Ideological and political education focuses on cultivating college students to establish a correct concept of life and improving their spiritual accomplishment, which to some extent promotes mental health education (Qian et al., 2018; Rogoza et al., 2018; Chen, 2019). However, most studies have separated the two aspects, ignoring the internal connection between them and the huge effect of their fusion. In view of this, an investigation is made on the intolerance of uncertainty, social anxiety, and negative emotions of college students through various methods including literature survey method, field investigation method, and intervention experiment method. Then, the intervention is implemented on the students by ideological and political education integrated with mental health education, obviously improving their negative emotions. This work is aimed at providing reference for improving the mental health education system of college students and strengthening the positive guidance of negative emotions of college students.

## Overview of Research-Related Definitions and Intervention Experiment Design

### Ideological and Political Education

The ideological and political education focuses on correct ideas conveyed to social groups, including ideological, political, and moral views. The ideological and political education affects the ideology of social groups, forming ideology and morality conforming to the social group development for social group members, through organized education activities (Hong, 2017). College students, as high-quality groups in society, need to grasp the dynamics of domestic political thoughts timely and form a good world outlook, outlook on life and values, focusing on a correct political outlook (Jorgenson et al., 2018; Haslam et al., 2019). China has put forward clear requirements for the training of talents in colleges and universities. It assumed that college education needs not only to guide students to form independent learning capabilities and smooth communication skills (Hong et al., 2018), but also to focus on the shaping of personality of students to promote good political literacy and moral qualities, considering their own characteristics. Ideological and political education is of great significance to the personality shaping of college students (Apanasyuk et al., 2019). Thus, colleges and universities need to guide the psychological development of students in accordance with domestic and foreign political situations and mainstream social thoughts and carry out targeted ideological and political education for students, to promote students to continuously improve and optimize their personal qualities, to meet the needs of social development and the progress of society.

The rich ideological and political education of college students contains the three outlooks education, moral education, legal education, and ideal and belief education (Boyle et al., 2020). The three outlooks include world outlook, outlook on life, and values. At present, the most scientific world outlook in the world is Marxist philosophy. Therefore, it is necessary to follow Marxist theory in the teaching of world outlook for college students. Moral education is the teaching of the moral concept of undergraduates, while legal education aims at improving students to meet the needs of national legal construction. Ideal and belief education is the “spiritual calcium” during the growth of college students, and the great energy and motivation for college students to participate in the construction of socialism with Chinese characteristics and contribute to the great rejuvenation of the Chinese nation. As a unique spiritual phenomenon of human beings, spiritual faith arises in certain material conditions and in turn guides the practical behavior of people.

### Mental Health Education

In 2018, the Ministry of Education issued *Guiding Outline of Mental Health Education for College Students* pointing out that, “Adhere to the unity of mental health education and moral education, strengthen humanistic care and psychological counseling, develop mental health education and advisory services normatively, and better adapt to and meet the needs of students’ mental health education services.” Mental health education is the theoretical system and method of multiple disciplines based on the combination of psychology and pedagogy (Blewitt et al., 2020), with planned and purposeful educational activities (Wu et al., 2019; Wu and Song, 2019). The mental health education of college students is based on the concept of “grand mental health education,” which refers to the practical activities reflected in all links and contents of education as the purpose and essence of education to help college students improve the healthy psychological basis and spiritual concepts including the scientific and objective ideological understanding, moral cultivation, ideal and belief (Mohr et al., 2017; de Oliveira Araújo et al., 2020). Mental health education covers aspects like psychological counseling, group counseling, psychological knowledge popularization, psychological crisis intervention, and mental health education activities. It also gradually integrates and cooperates with other education during development, constantly enhancing its value and function.

Mental health education is the fourth subsystem of modern ideological and political education (Kharkovskaya et al., 2017), besides traditional ideological and political education (ideological education, political education, and moral education). The relationships between mental health education and traditional ideological and political education and with the grand ideological and political education system have become the theoretical and practical focus of modern ideological and political educators (Stein et al., 2019).

### Negative Emotions of College Students

Emotion is a comprehensive physiological and psychological state of the behavior and subjective feelings of a person caused by external stimuli. It is initially such psychological reactions as joy, anger, and sorrow and then produces changes in hormones in the body, accompanied by physiological reactions including changes in hormone levels and sweating (Thompson, 2019). Negative emotion is excessive reaction of a person to objective stimulus, containing anxiety, tension, anger, depression, grief, pain, sadness, unhappiness, and depression (De Vaus et al., 2018; Young et al., 2019). Such emotions have negative effects on the body, work, and life. College students suffer more frequently from negative emotions than other groups, therefore needing special attention. Furthermore, emotions are closely contacted with cognition. Studies have shown that negative emotions can cause attention problems and memory decline.

From the perspective of psychological characteristics, some domestic experts define emotion regulation as a process of self-evaluation of cognition and an essential psychological condition to assist people to achieve emotional changes (Garber et al., 2019). Other studies assume that emotion regulation is the process of individuals exerting influence on the occurrence, experience, and expression of emotions, thereby regulating, inhibiting, or enhancing emotional experience and expression (Parise et al., 2019). In short, emotion regulation includes one or more combined strategies used by people to enhance, maintain, or reduce all consciously or unconsciously changing emotions. It can monitor, judge, evaluate, and change the psychology and external behavior process of all emotional reactions (Smith et al., 2019).

### Design of Intervention Experiment

(1) Experimental object: Sophomores and juniors in a university are taken as experimental objects, who are from two classes in the two grades, with the same teachers in the two parallel classes. Among the two classes in each grade, one is randomly selected as the treatment group and the other as the control group.
(2) Experimental hypothesis: Ideological and political education integrated with mental health education positively impacts the negative emotions of college students.
(3) Irrelevant variable control: The teachers in the treatment group and the control group are the same. Considering the universal preventive nature of the intervention methods used, the objects are not screened in terms of mental health status, drug treatment history, and psychiatric history before the experiment. For the better ecological validity of the experiment by using a school as a platform and a class as a unit in practice, a natural class of the school serves as a unit without random grouping of experimental objects.

## Results and Analysis

### Descriptive Analysis of Samples

**FIGURE 1:**
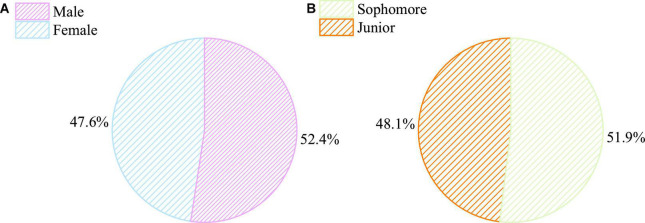
Basic information of subjects. **(A)** Gender distribution. **(B)** Grade distribution.

The basic information of subjects is shown in Figure 1.

Ninety-five electronic questionnaires are distributed to students in a university by handy sampling, receiving 93 back with a recovery of 97.89%. Among them are 5 invalid and 88 effective questionnaires, with an effective recovery of 94.62%. A quality test was also conducted on the questionnaires by SPSS 22.0 software to evaluate the α coefficient of reliability to verify the reliability, stability, and indication system of the data and prepare for the next testing.

### Comparison Between the Treatment Group and Control Group Before the Experiment

**FIGURE 2:**
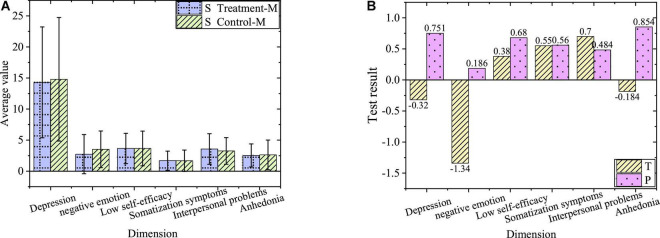
Comparison of negative emotions of sophomores. **(A)** Average of treatment group and control group. **(B)** Value of T and P of treatment group and control group.

The comparison of negative emotions between the treatment group and control group in sophomore year before the experiment is shown in Figure 2.

Figure 2 shows the overall negative emotion level of the sophomore treatment group and control group and *P* values of each dimension level greater than 0.05. Accordingly, there is no significant difference in negative emotions and the levels of each dimension between sophomores in the treatment group and control group before the experiment.

**FIGURE 3:**
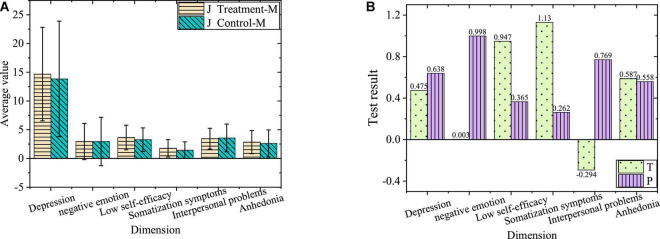
Comparison of negative emotions of juniors. **(A)** Average of treatment group and control group. **(B)** Value of T and P of treatment group and control group.

The comparison of negative emotions between the junior treatment group and control group before the experiment is shown in Figure 3.

Figure 3 shows the overall negative emotion level of the junior treatment group and control group and *P* values of each dimension level greater than 0.05. Accordingly, there is no significant difference in negative emotions and the levels of each dimension between juniors in the treatment group and control group before the experiment. During the two experimental groups, the negative emotion level of the experimental subjects, the control mode of irrelevant variables, and the intervention methods of the treatment group maintain the same. Therefore, the subjects are divided into the treatment group and control group irrespective of grades.

**FIGURE 4:**
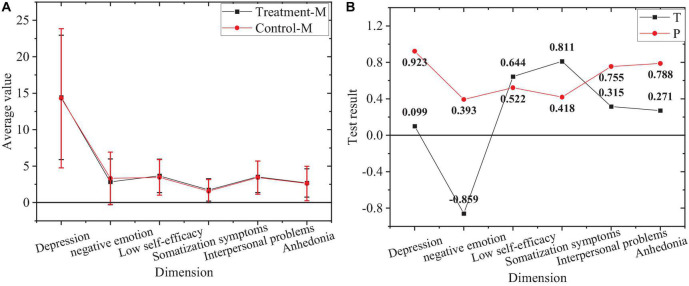
Comparison of negative emotions. **(A)** Average of treatment group and control group. **(B)** Value of T and P of treatment group and control group.

The comparison of negative emotions between the treatment group and control group before the experiment is shown in Figure 4.

Figure 4 denotes the overall negative emotion level of the treatment group and control group and *P* values of each dimension level greater than 0.05. Accordingly, there is no significant difference in negative emotions and the levels of each dimension between the treatment group and control group before the experiment.

**FIGURE 5:**
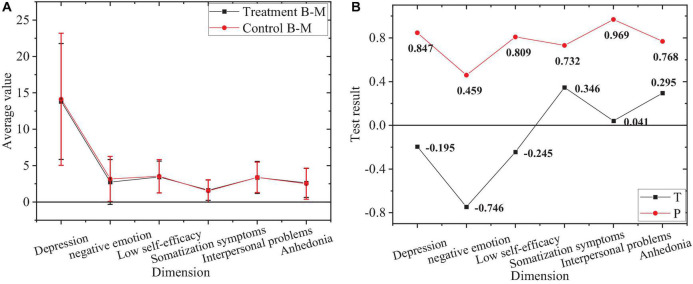
Comparison of negative emotions. **(A)** Average of male subjects in the treatment group and control group. **(B)** Value of T and P of male subjects in the treatment group and control group.

The comparison of negative emotions between male subjects before the experiment is shown in Figure 5.

The two groups of subjects are separated according to their genders to test whether there is a significant difference in the level of negative emotions between the same gender in the treatment group and control group before the experiment. Figure 5 shows the negative emotions of male subjects in the treatment group and control as well as the *P* values of all dimensions greater than 0.05. Therefore, there is no significant difference in negative emotions and the levels of each dimension between male subjects in the treatment group and control group before the experiment.

The comparison of negative emotions between female subjects before the experiment is shown in Figure 5.

**FIGURE 6:**
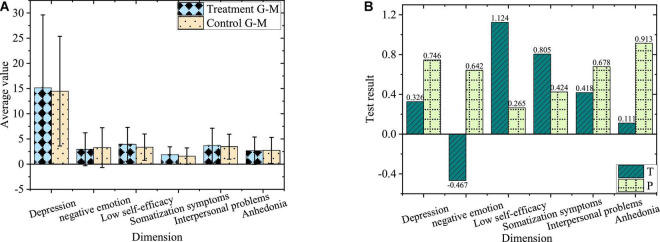
Comparison of negative emotions. **(A)** Average of female subjects in treatment group and control group. **(B)** Value of T and P of female subjects in treatment group and control group.

Figure 6 shows the negative emotions of female subjects in the treatment group and control as well as the *P* values of all dimensions greater than 0.05. Thus, there is no significant difference in negative emotions and the levels of each dimension between female subjects in the treatment group and control group before the experiment.

### Comparison of Negative Emotions of the Treatment Group Before and After the Experiment

**FIGURE 7:**
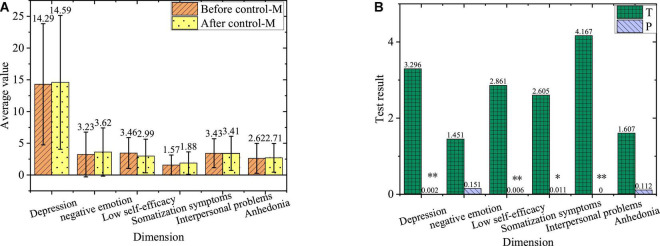
Comparison of negative emotions. **(A)** The treatment group before and after the experiment. **(B)** Value of T and P of the treatment group before and after the experiment. *Denotes a significant difference, while ^**^ represents a highly significant difference.

The comparison of negative emotions between subjects of the treatment group before and after the experiment is shown in Figure 7.

Clearly, after the intervention of ideological and political education integrated with mental health education for 10 weeks, the students in the treatment group have highly significant changes in depression, self-efficacy, somatization symptoms, and interpersonal problems. Although there is no significant change in negative emotion and anhedonia, there is a tendency to improve their dimensions. Therefore, ideological and political education integrated with mental health education can effectively improve the negative emotional levels of college students.

**FIGURE 8:**
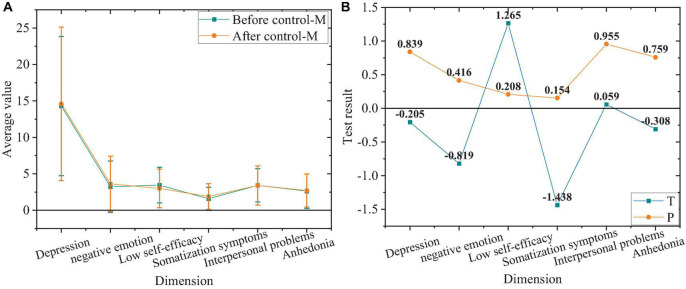
Comparison of negative emotions. **(A)** The control group before and after the experiment. **(B)** Value of T and P of the control group before and after the experiment.

The comparison of negative emotions between subjects of the control group before and after the experiment is shown in Figure 8.

Figure 8 indicates that the negative emotion level and each dimension level of the control group without the intervention of ideological and political education integrated with mental health education does not change significantly after the experiment compared with those before the experiment. That is, the negative emotion level of the control group does not change after the experiment.

### Comparison Between the Treatment Group and Control Group After the Experiment

**FIGURE 9:**
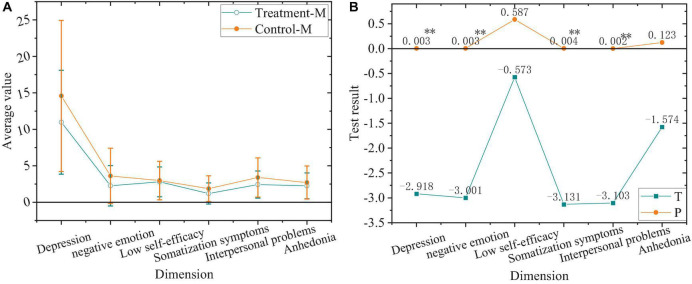
Comparison of negative emotions. **(A)** Values of the treatment group and control group after the experiment. **(B)** Value of T and P of the treatment group and control group after the experiment. “*”Means a significant difference, while “^**^” signifies a highly significant difference.

The comparison between the treatment group and control group after the experiment is shown in Figure 9.

In Figure 9, students in the treatment group and control group after the experiment have a highly significant difference in depression, negative emotion, somatization symptoms, and interpersonal problems (*P* < 0.01), with no significant difference in the low self-efficacy and anhedonia (*P* > 0.05).

**FIGURE 10:**
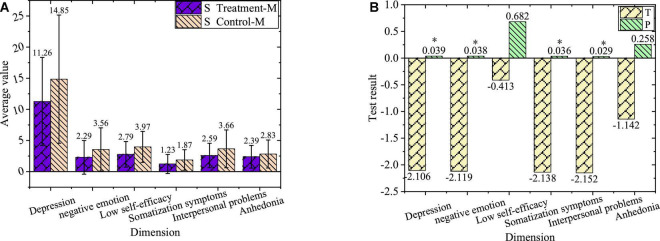
Comparison of negative emotions. **(A)** Values of sophomores after the experiment. **(B)** Value of T and P of sophomores after the experiment. “*”Refers to a significant difference, while “^**^” represents a highly significant difference.

The comparison of negative emotions of sophomore treatment group and control group after the experiment is shown in Figure 10.

In Figure 10, the sophomores in the treatment group and control group after the experiment have a significant difference in depression, negative emotion, somatization symptoms, and interpersonal problems (*P* < 0.01), with no significant difference in low self-efficacy and anhedonia (*P* > 0.05).

**FIGURE 11:**
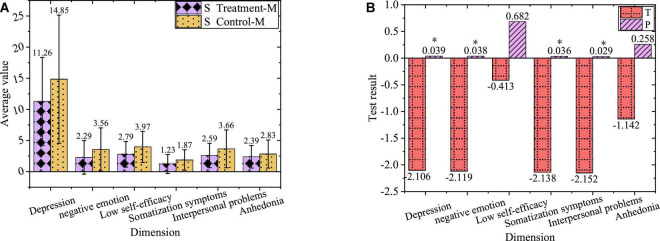
Comparison of negative emotions. **(A)** Values of juniors after the experiment. **(B)** Value of T and P of juniors after the experiment. “*”Means a significant difference, while “^**^” shows a highly significant difference.

The comparison of negative emotions of junior treatment group and control group after the experiment is shown in Figure 11.

In Figure 11, the junior treatment group and control group after the experiment have a highly significant difference in depression, negative emotion, somatization symptoms, and interpersonal problems (*P* < 0.01), with no significant difference in low self-efficacy and anhedonia (*P* > 0.05).

**FIGURE 12:**
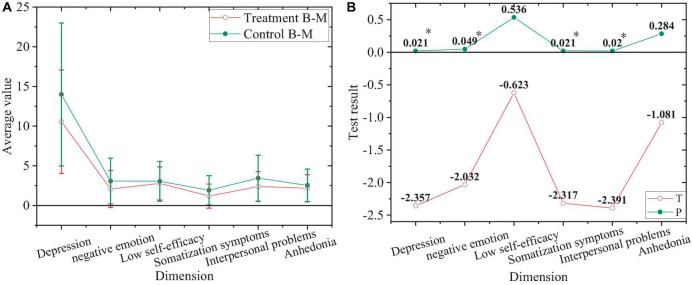
Comparison of negative emotions. **(A)** Values of male subjects after the experiment. **(B)** Value of T and P of male subjects after the experiment. “*”Shows a significant difference, while “^**^” means a highly significant difference.

The comparison of negative emotions between male subjects in the treatment group and control group is shown in Figure 12.

In Figure 12, the male subjects in the treatment group and control group after the experiment have a significant difference in depression, negative emotion, somatization symptoms, and interpersonal problems (*P* < 0.01), with no significant difference in low self-efficacy and anhedonia (*P* > 0.05).

**FIGURE 13:**
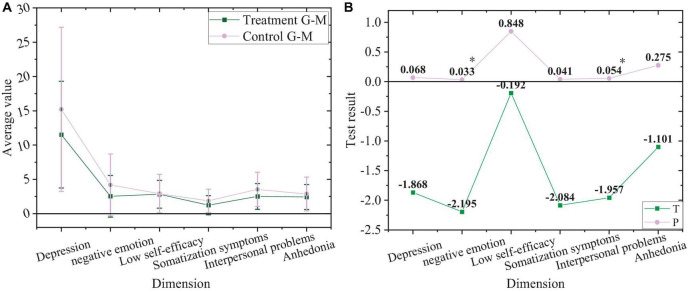
Comparison of negative emotions. **(A)** Values of female subjects after the experiment. **(B)** Value of T and P of female subjects after the experiment. “*”Represents a significant difference, while “^**^” shows a highly significant difference.

The comparison of negative emotions between female subjects in the treatment group and control group after the experiment is shown in Figure 13.

In Figure 13, the female subjects in the treatment group and control group after the experiment have no significant difference in depression, low self-efficacy, interpersonal problems, and anhedonia (*P* > 0.05), with a significant difference in negative motion and somatization symptoms (*P* < 0.01).

In this experiment, the combination of ideological and political education and mental health education is used to guide college students to resist negative emotions. In summary, compared with the control group, the treatment group has been significantly improved in depression, negative emotions, somatization symptoms, and interpersonal problems. Among them, the improvement of the psychological state of male subjects is more obvious, but the experimental results of female subjects are not significant. The reason may be that male subjects have stronger self-regulation ability, and they can recover quickly after external intervention. On the contrary, the self-psychological adjustment ability of female subjects is slightly poor. Consequently, in the intervention treatment, the psychological state of female subjects may not be effectively regulated in a short period.

## Conclusion

Nowadays, ideological and political education has a relatively complete system, and it focuses on cultivating college students to establish a correct concept of life and guiding students to resist negative emotions. Here, ideological and political education is combined with mental health education to verify the effects of collaborative intervention on guiding college students to resist negative emotions. A comprehensive introduction is first presented of the ideological and political education, mental health education, and negative emotions of college students by literature study, under the condition of the negative emotions problems of college students to be solved. Then, an experiment is taken on college students combined with ideological and political education integrated with mental health education, after the overview of the emotional status of contemporary college students. Finally, the negative emotions of the students are compared before and after the experiment. The experimental results indicate that after 10 weeks of intervention experiment by combining ideological and political education with mental health education, there are significant differences in depression, negative emotions, somatization symptoms, and interpersonal problems between the treatment group and the control group (*P* < 0.01). Specifically, there are significant differences in depression, negative emotions, somatization symptoms, and interpersonal problems between sophomores in the treatment group and the control group (*P* < 0.01). Besides, there are significant differences in depression, negative emotions, somatization symptoms, and interpersonal problems between male subjects in the treatment group and the control group (*P* < 0.01). In conclusion, ideological and political education integrated with mental health education can improve the negative emotions of undergraduates and reduces their pressure of negative emotions largely to enjoy a more relaxed and active learning life. Despite the comprehensive experiment and the effective method, the teaching intervention is limited due to objective reasons. The education scope is expected to be expanded to continuously improve the experiment. The research results can facilitate the optimization of mental health education of college students, ameliorating negative emotions of college students.

## Data Availability Statement

The raw data supporting the conclusions of this article will be made available by the authors, without undue reservation.

## Ethics Statement

The studies involving human participants were reviewed and approved by Guangzhou Civil Aviation College Ethics Committee. The patients/participants provided their written informed consent to participate in this study. Written informed consent was obtained from the individual(s) for the publication of any potentially identifiable images or data included in this article.

## Author Contributions

All authors listed have made a substantial, direct, and intellectual contribution to the work, and approved it for publication.

## Conflict of Interest

The authors declare that the research was conducted in the absence of any commercial or financial relationships that could be construed as a potential conflict of interest.

## Publisher’s Note

All claims expressed in this article are solely those of the authors and do not necessarily represent those of their affiliated organizations, or those of the publisher, the editors and the reviewers. Any product that may be evaluated in this article, or claim that may be made by its manufacturer, is not guaranteed or endorsed by the publisher.
